# Effects of *Lactococcus lactis* subsp. *cremoris* YRC3780 daily intake on the HPA axis response to acute psychological stress in healthy Japanese men

**DOI:** 10.1038/s41430-021-00978-3

**Published:** 2021-08-04

**Authors:** Noriko Matsuura, Hidemasa Motoshima, Kenji Uchida, Yujiro Yamanaka

**Affiliations:** 1 Laboratory of Life & Health Sciences, Faculty of Education and Graduate School of Education, Hokkaido University, Sapporo, Japan; 2 R&D Center, Yotsuba Milk Products Co., Ltd, Kitahiroshima, Japan; 3 Research and Education Center for Brain Science, Hokkaido University, Sapporo, Japan

**Keywords:** Randomized controlled trials, Sleep

## Abstract

**Background:**

*Lactococcus lactis* subsp. *cremoris* (YRC3780), which is isolated from kefir, has been associated with anti-allergic effects in humans. However, it remains unknown whether daily intake of YRC3780 attenuates the response to psychological stress in humans in parallel with changes to the gut microbiome. We examined the fundamental role of YRC3780 in the gut microbiome, stress response, sleep, and mental health in humans.

**Methods:**

Effects of daily intake of YRC3780 on the hypothalamic-pituitary-adrenal (HPA) axis response to acute psychological stress were investigated in a double-blind, placebo-controlled clinical trial involving 27 healthy young men (mean age and body mass index: 23.5 years and 21.5 kg/m^2^) who were randomly assigned to placebo (*n* = 13) or YRC3780 (*n* = 14) groups. The HPA axis response to acute psychological stress, the diurnal rhythm of HPA axis activity, and gut microbiome were assessed and compared between the two groups.

**Results:**

The results showed that daily intake of YRC3780 significantly lowered morning salivary cortisol levels compared with placebo. In addition, salivary cortisol levels following a social stress test significantly decreased +40 min after beginning the TSST in the YRC3780-treated group compared to placebo. There were no significant differences between the two groups in terms of actigraphy-based sleep quality, but the subjective sleep quality and mental health were significantly improved in the YRC3780-treated group compared to placebo.

**Conclusions:**

Our study suggests that daily intake of YRC3780 improves the HPA axis response to acute psychological stress, which might be associated with a decrease in morning cortisol levels.

## Introduction

The hypothalamic-pituitary-adrenal (HPA) axis and sympathetic adrenomedullary (SAM) systems are major components of the stress response system [1]. When individuals are exposed to acute psychological stress, activation of the HPA axis and SAM system increase glucocorticoid hormone (cortisol) levels and heart rate (HR) [1, 2]. This HPA axis stress response is influenced by circadian rhythms [3, 4], sleep [5, 6], and psychological stress [7, 8].

Recent research advancements have clarified the relationships between gut microbiota functions and metabolic syndrome, autism, autoimmunity, and cancer [9]. Several studies have focused on the relationship between brain function and intestinal microorganisms [10–12]. In rodents, HPA stress responses were suppressed in specific pathogen-free mice compared with germ-free mice, suggesting that the suppressive effects of the stress response on the gut microbiota depend on the bacterial species [13]. In humans, several studies on intestinal microorganisms and stress responses have confirmed the efficacy of probiotic intake [14–19], and the effectiveness of killed bacteria [20]. An alternative mechanism involved in the alteration of the gut microbiota and/or the efficacy of probiotics on the stress response may be associated with the so-called gut-brain axis, a bidirectional interaction between the gut microbiome and the central nervous system [21]. As for the afferent pathways from the gut to the brain, tryptophan, and mainly its metabolite serotonin, is released from intestinal chromaffin cells by intestinal bacteria. Serotonins act on the 5-HT3 receptor present on the vagus nerve or on spinal cord salvage nerve terminals and signal to the brain via the solitary nucleus. Neural substrates metabolized by intestinal bacteria such as short-chain fatty acids, immune cells (cytokines), and GABA directly or indirectly affect the brain [10, 21–23].

Studies have confirmed that anti-inflammatory cytokines and regulatory T cells work effectively in animal models of multiple sclerosis (MS) and in experimental autoimmune encephalomyelitis [24, 25], which are neuroinflammatory diseases of the central nervous system affecting brain and spinal cord function. Based on these findings, lactic acid bacteria that act on immune cells, even if they are not probiotics, may affect the stress response. *Lactococcus lactis* subsp. *cremoris* YRC3780 (hereafter ‘YRC3780’), which is isolated from kefir, has been shown to exert anti-allergic effects in humans [26] and immunostimulatory effects, and induces Foxp3+regulatory T cell activity confirmed in an animal study [27]. However, it remains unknown whether YRC3780 affects the human stress response and the gut microbiome. The present study assessed the effects of daily YRC3780 intake on subjective and objective sleep quality, mental health, basal activity of the HPA axis, and stress reactivity in healthy young male participants.

## Materials and methods

### Ethical approval

All study protocols were approved by the ethical committee of Hokkaido University Graduate School of Education (no. 17–41) and conducted according to the Declaration of Helsinki.

### Participants

Twenty-seven healthy young male participants (mean age 23.5 years and body mass index 21.5 kg/m^2^) completed the study as paid volunteers. Table 1 summarizes the placebo and YRC3780 participant characteristics. Participants were recruited through advertisements at Hokkaido University in Japan. The enrollment criteria included subjects who did not have jobs that required early morning, late night, or rotating night shifts, and none had a personal history of psychiatric, endocrine, or sleep disorders. All participants provided written informed consent prior to entering the study, which allowed them to withdraw from the experiment at any time.

**Table 1:** Descriptive statistics by group.

	Placebo (*n* = 13)	YRC3780 (*n* = 14)	*P* value
Age (years)	23.2 ± 3.8	23.8 ± 5.1	0.952
Height (cm)	169.8 ± 6.2	170.7 ± 6.1	0.592
Body weight (kg)	61.0 ± 7.5	63.4 ± 7.5	0.232
Body mass index (kg/m^2^)	21.8 ± 2.6	21.1 ± 2.1	0.765
PSQI score	4.0 ± 1.8	4.4 ± 1.6	0.613
AIS score	4.3 ± 2.4	3.8 ± 2.4	0.604
GHQ-28 score	4.3 ± 3.2	3.7 ± 3.2	0.460
POMS 2 TMD score	46.3 ± 5.1	48.3 ± 7.8	0.573

Values are means ± SD*.*

*PSQI* Pittsburgh Sleep Quality Index, *AIS* Athens Insomnia Scale, *GHQ-28* General Health Questionnaire, *POMS 2 TMD* Profile of Mood States 2nd Edition, Total Mood Disturbance subscale.

### Experimental protocol

**Fig. 1:**
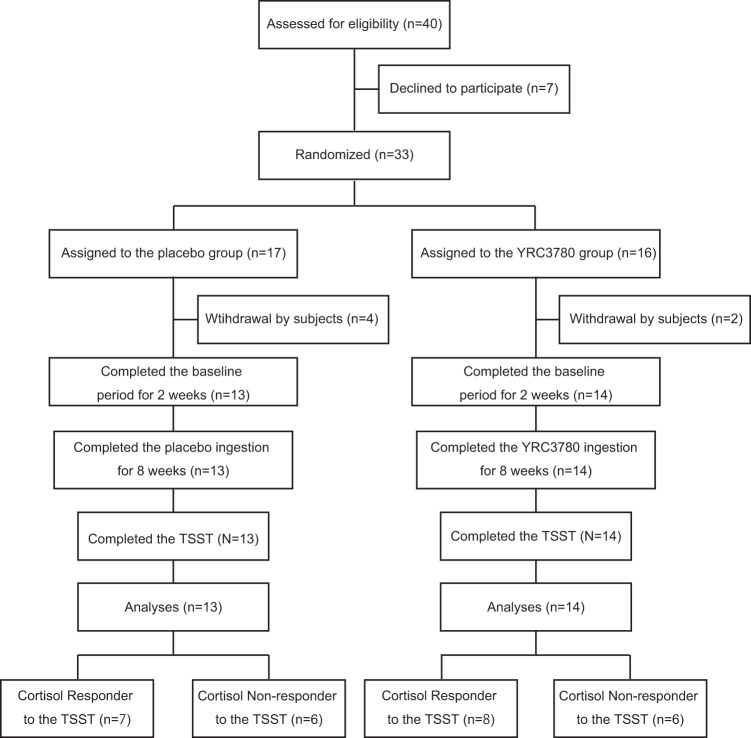
Experimental protocol. Study design and flow chart of the double-blind, placebo-controlled, and randomized trial in healthy Japanese men.

This double-blind and placebo-controlled clinical trial was conducted throughout 2018 and 2019. Figure 1 shows participant flow throughout the study, which involved a baseline period of 2 weeks and daily ingestion of placebo or YRC3780 capsules for 8 weeks. Participants wore a wrist actigraphy sensor and kept a daily diary throughout the experiment to collect data on bedtimes, wake times, subjective sleep, mealtimes, and capsule ingestion times. To evaluate sleep quality, mood, and general health status, each participant completed the Athens Insomnia Scale (AIS) [28, 29], Pittsburgh Sleep Quality Index (PSQI) [30, 31], General Health Questionnaire (GHQ-28) [32], and Profile of Mood States 2nd Edition-Adult Short, Total Mood Disturbance subscale (POMS 2 TMD) [33] every 2 weeks throughout the study (starting at the last day of baseline, and 2, 4, 6, and 8 weeks after beginning capsule ingestion). The AIS score ranges from 0 to 28: scores of 0–3 are classified as no insomnia, scores of 4–5 are suggestive of insomnia, and scores of ≥6 are strongly suggestive of insomnia [28, 29]. The PSQI score ranges from 0 to 21: a lower score indicates better sleep quality and a higher score (≥6) indicates poor sleep quality [30, 31]. The GHQ-28 score ranges from 0 to 28: <5 is classified as good mental health (better than usual) and a higher score indicates psychiatric distress (worse than usual) [32]. The POMS 2 TMD score ranges from 30 to 70 where scores 40–59 classify the normal mood state, while ≥60 indicates higher mood disturbance (unfavorable psychological state) [33]. To assess HPA axis activity diurnal rhythms, every 2 weeks participants provided saliva samples collected at 2 h intervals throughout the day, starting immediately after waking. In addition, three fecal samples were collected to analyze the gut microbiome (on the last day of baseline, and at 4 and 8 weeks after beginning capsule ingestion). Within 1 week after completing the 8-week capsule ingestion period, the participants completed the Trier Social Stress Test (TSST) at the laboratory to assess the effect of daily YRC3780 ingestion on the HPA axis stress response in a laboratory setting.

The Supplementary data includes information regarding the microbiological characterization of YRC3780, the test meal, the TSST protocol, measurement of salivary cortisol levels, microbiome analysis, PCR primer (Table S1), and statistical analysis in the present study.

## Results

### Diurnal salivary cortisol rhythms

**Fig. 2:**
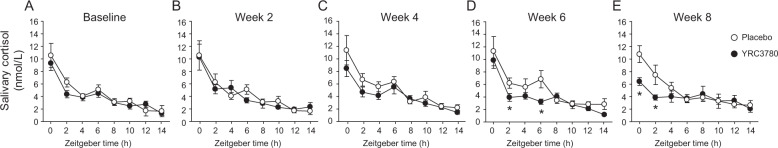
Diurnal rhythm of salivary cortisol in the placebo and YRC3780 groups. Mean diurnal rhythm of salivary cortisol concentration measured at baseline (**A**) and weeks 2 (**B**), 4 (**C**), 6 (**D**), and 8 (**E**) of the placebo and YRC3780 ingestion period. Data are expressed as the mean ± SEM (placebo, *n* = 13; YRC3780, *n* = 14). *p < 0.05 vs. placebo using the Mann-Whitney U test.

Figure 2 illustrates the diurnal salivary cortisol rhythms measured on the last day of the 2-week baseline period and every 2 weeks during the 8-week ingestion period. Friedman tests identified significant changes in diurnal rhythms in both the YRC3780 and placebo groups during the experiment. On the last day of the baseline period and at week 2 of the ingestion period, salivary cortisol levels were not significantly different between the placebo and YRC3780 groups (Fig. 2A,B). After 4 weeks of ingestion, the morning salivary cortisol levels (0 h and 6 h after waking) were slightly decreased in the YRC3780 group compared with the placebo group (Fig. 2C). At week 6 of YRC3780 intake, salivary cortisol levels at 2 h and 6 h after waking were significantly lower than those in the placebo group (Fig. 2D). The lowered salivary cortisol levels in the YRC3780 group persisted at week 8 of the ingestion period, with salivary cortisol levels measured at 0 h and 2 h after waking significantly lower in the YRC3780 group than in the placebo group (Fig. 2E).

### HPA axis and sympathetic nervous system response to the TSST

**Fig. 3:**
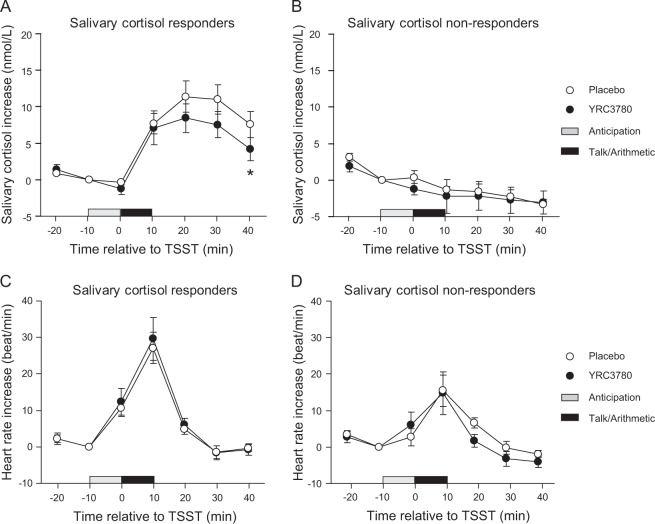
Salivary cortisol concentration and heart rate responses before and after the Trier Social Stress Test (TSST) in the placebo and YRC3780 groups. Data are expressed as mean ± SEM. **A** and **C** Indicate the salivary cortisol and heart rate increases in the salivary cortisol responders (placebo, *n* = 7; YRC3780, *n* = 8), while (**B**) and (**D**) indicate these values in the non-responders (placebo, *n* = 6; YRC3780, *n* = 6). Open and closed circles indicate data from the placebo and YRC3780 groups, respectively. Grayed and closed horizontal bars indicate 10 min anticipation and 10 min TSST period. *p < 0.05 vs. placebo using the Mann–Whitney U test.

To evaluate the effects of daily placebo/YRC3780 ingestion on the HPA axis stress response, participants were categorized as salivary cortisol responders and non-responders based on previously published classification criteria (1.5 nmol/L or 15.5% increase) [34]. The classification resulted in 15 cortisol responders (placebo group, *n* = 7; YRC3780 group, *n* = 8) and 12 cortisol non-responders (placebo group, *n* = 6; YRC3780 group, *n* = 6). In the cortisol responders, the salivary cortisol concentration in the YRC3780 group was lower after the TSST than in the placebo group (Fig. 3A). Salivary cortisol concentrations at 40 min after the TSST were significantly lower in the YRC3780 group (4.2 ± 4.4 nmol/L) (mean ± SD) than in the placebo group (7.6 ± 4.7 nmol/L) (*p* = 0.043, Mann–Whitney U test), while salivary cortisol concentrations in the non-responders did not show a significant increase after the TSST.

Although the salivary cortisol concentrations after the TSST in the cortisol responders were significantly different between the placebo and YRC3780 groups, the HR after the TSST did not differ significantly between the two groups (Fig. 3C). Interestingly, the salivary cortisol concentrations in the non-responders did not significantly increase after the TSST (Fig. 3B), whereas the HR after the TSST increased to the same extent in both the placebo and YRC3780 groups (Fig. 3D).

### Questionnaire and sleep parameters

Table 2 summarizes the questionnaire results for the baseline and 8-week placebo/YRC3780 ingestion period. During the ingestion period, the AIS scores at 6 weeks and GHQ-28 scores at 8 weeks were significantly lower in the YRC3780 group than in the placebo group (AIS, *p* = 0.031; GHQ-28 *p* = 0.038, Mann–Whitney U test). Table 3 summarizes the results of the actigraphy-measured sleep efficiency for the baseline and 8-week placebo/YRC3780 ingestion period. There were no significant differences between the two groups at any time point.

**Table 2:** Subjective sleep quality, mental health, and mood state before and during the ingestion period.

		Ingestion period (weeks)				
		Baseline	2	4	6	8
PSQI	Placebo	4.0 ± 1.8	4.0 ± 2.1	4.3 ± 2.0	3.8 ± 2.2	3.8 ± 2.0
	YRC3780	4.4 ± 1.6	4.0 ± 1.8	4.2 ± 1.5	3.3 ± 1.6	3.5 ± 1.2
	*P* value	0.613	0.953	0.917	0.815	0.881
AIS	Placebo	4.3 ± 2.4	4.4 ± 2.5	4.2 ± 2.8	3.8 ± 2.0	4.1 ± 2.6
	YRC3780	3.8 ± 2.4	3.6 ± 1.5	3.8 ± 2.6	2.1 ± 1.1^a^	2.6 ± 1.5
	*P* value	0.604	0.759	0.771	0.031	0.134
GHQ-28	Placebo	4.3 ± 3.2	3.1 ± 2.6	3.7 ± 3.5	3.1 ± 4.2	3.5 ± 4.0
	YRC3780	3.7 ± 3.2	2.7 ± 2.6	3.2 ± 3.6	2.5 ± 1.7	1.4 ± 1.4^a^
	*P* value	0.460	0.617	0.503	0.538	0.038
POMS 2 TMD	Placebo	46.3 ± 5.1	46.3 ± 9.4	43.2 ± 7.7	44.4 ± 10.3	43.1 ± 6.5
	YRC3780	48.3 ± 7.8	45.4 ± 3.6	44.5 ± 5.9	43.0 ± 6.1	42.6 ± 4.2
	*P* value	0.573	0.622	0.330	0.952	0.972

Values are means ± SD (*n* = 12–14). The *p* values in columns represent comparisons between YRC3780 and placebo by the Mann–Whitney U test*.*

*PSQI* Pittsburgh Sleep Quality Index, *AIS* Athens Insomnia Scale, *GHQ-28* General Health Questionnaire, *POMS 2 TMD* Profile of Mood States 2nd Edition, Total Mood Disturbance subscale.

The *p* values represent comparisons between YRC3780 and placebo by Mann-Whitney U test.

^a^*p* < 0.05 vs. placebo.

**Table 3:** Actigraphy-measured sleep efficiency before and during the ingestion period.

	Ingestion period (week)				
	Baseline	2	4	6	8
Placebo	87 ± 8	87 ± 7	87 ± 8	87 ± 7	86 ± 8
YRC3780	83 ± 5	84 ± 7	83 ± 6	83 ± 5	84 ± 6
*P* value	0.139	0.264	0.198	0.068	0.242

Data are expressed as means ± SD. Sleep efficiency was defined as the ratio of TST to SPT as a percentage. The SPT was defined as the length of the sleep interval from the first epoch counted as sleep to the last epoch counted as sleep in the main sleep interval. TST was defined as the total number of minutes counted as sleep in the main sleep interval, in hours. The *p* values represent comparisons between YRC3780 and placebo by the Mann–Whitney U test.

### Fecal microbiome

**Fig. 4:**
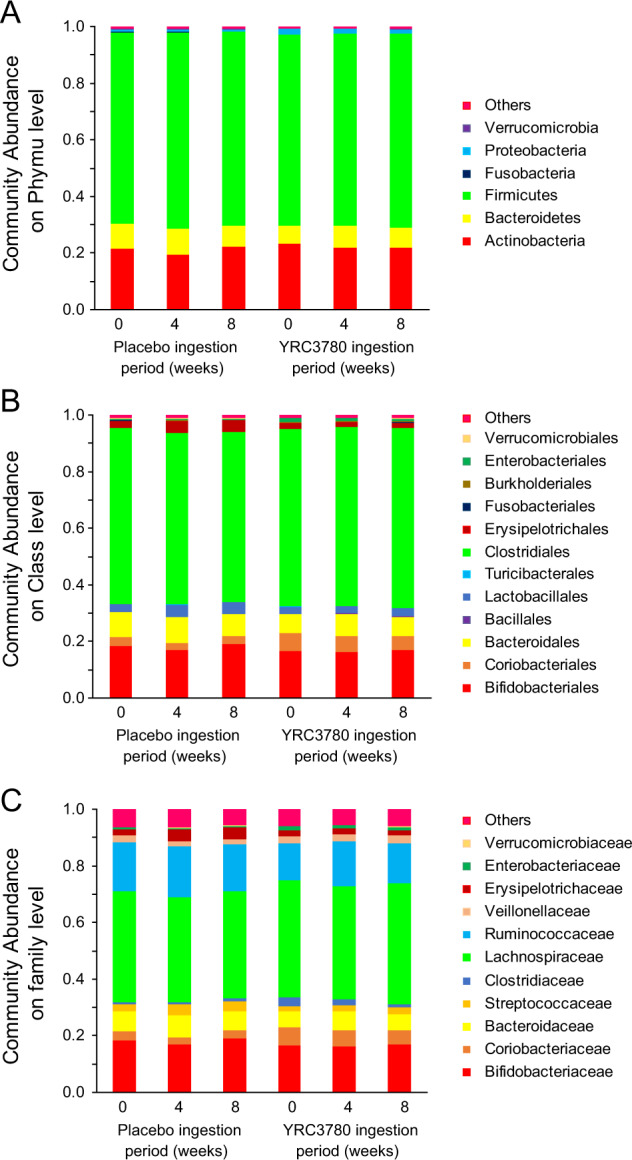
Gut microbiota composition of test meal intake group and placebo meal intake group. Abundant phyla (**A**), families (**B**), and genera (**C**) in the gut microbiota from the placebo and YRC378 groups. Only families and genera with average relative abundance >1% are shown. Data are the mean values (placebo, *n* = 13; YRC3780, *n* = 14).

Alpha diversity (Chao 1, Shannon) and fecal microbiota analysis was performed on all 81 fecal samples collected from the participants on the last day of baseline evaluations, and at 4 and 8 weeks after capsule ingestion. First, to assess the effects of the fecal microbiome on the HPA axis response to the TSST, the placebo and YRC3780 group participants were divided into cortisol responders and non-responders based on the published classification [34], and intra- and inter-group differences were compared. There were no significant differences between the responders and non-responders; therefore, all data in each group were averaged and used in the group comparisons. Figure 4 shows the results of the fecal microbiota analysis in the placebo and YRC3780 groups.

## Discussion

We demonstrated that daily intake of YRC3780 decreased morning salivary cortisol levels 6 and 8 weeks after beginning daily ingestion (Fig. 2) and decreased the salivary cortisol response to acute psychological stress induced by the TSST (Fig. 3). Basal activity of the HPA axis and its stress response exhibited a 24 h circadian rhythm with higher activity in the morning than in the afternoon and evening [3, 4]. Thus, the decrease in the cortisol stress response after TSST in the YRC3780 group might be associated with lower morning cortisol concentrations associated with daily ingestion of YRC3780. In turn, the daily intake of YRC3780 decreased the basal activity of the HPA axis and the stress response, which have been reported to be influenced by subjective and/or objective sleep quality [5, 6] and psychological stress [7, 8]. Although objective sleep quality measured by actigraphy was not significantly influenced by daily YRC3780 ingestion, subjective sleep quality measured by the AIS score decreased at 6 weeks of ingestion compared with the placebo group (Table 2). In addition, the YRC3780 group had a lower GHQ-28 score at 8 weeks of ingestion (Table 2). Lower AIS and GHQ-28 scores indicate better sleep quality and a better mental state, respectively. These results suggest that lower basal activity and the stress reactivity of the HPA axis in the YRC3780 group was associated with improved subjective sleep quality and mental health.

Regarding the HR stress reactivity to the TSST, both the cortisol responder and non-responder groups showed a similar tendency, for an increased HR just after starting the TSST. Nevertheless, the cortisol stress levels following the TSST were markedly different between cortisol responders and non-responders (Fig. 3). Both the HPA axis and SAM systems are major physiological responses to various stressors. These two stress response systems activate different brain networks and reaction times [1]. In the first phase of the stress response, the SAM system is rapidly activated, and HR is increased just after exposure to stressors. In the second phase, the HPA axis is activated and glucocorticoid hormone is secreted. The HPA axis stress reaction is influenced and modulated by various factors such as age, sex, early life environment, genetic factors, and HPA basal activity [1–4]. Thus, the rapid stress response pathway, the SAM system, might respond independently of the HPA axis response or may be more sensitive to stressors as compared with the HPA axis.

The gut microbiota has been reported to undergo changes, with improvement in the intestinal environment even after ingestion of heat-killed lactic acid bacteria [35–37]. In the present study, the daily intake of YRC3780 and placebo had no effect on the fecal microbiota throughout the experiment, which provides further evidence supporting the instability of YRC3780 in the presence of low pH and bile salts, and that it cannot reach the intestines alive (data not shown). Therefore, the lack of impact of YRC3780 on the fecal microbiota is consistent with previous observations. Although the specific bacterial species affecting the HPA axis have not yet been identified, changes in the composition of the intestinal bacteria may potentiate the basal activity of the HPA axis and the reactivity to stress. Indeed, short-chain fatty acids such as butyric acid produced by intestinal bacteria are considered a factor in the HPA axis stress response [22]. However, in the present study, the fecal microbiota and butyric acid content in the feces did not change (data not shown), suggesting that short-chain fatty acids did not contribute to our finding of lower cortisol concentrations in the YRC3780 group.

The immune system is also a factor in the stress response. Chronic stress, such as achieved via stress promotion tests, causes a decrease in natural killer (NK) cell activity, a tendency toward Th2 cell dominance [38–40], and NK in acute stress such as video speech. An increase in cytotoxic T cells (CD8 + T cells) and a decrease in regulatory T cells levels [40–43] have also been confirmed. In addition, inflammatory cytokines have been reported to exacerbate depression by attenuating serotonin production [39] and reducing regulatory T cell activity [44]. Thus, it is possible that the immune system and the stress response are closely associated with changes in the basal activity of the HPA axis and the stress response induced in the YRC3780 group. Moreover, YRC3780 enhances NK cell activity, which promotes IL-2 production in colon cancer-bearing mice [27]. In addition, YRC3780 modifies the Th1/Th2 balance and promotes regulatory T cell induction in ovalbumin-sensitized mice. Furthermore, a previous study in patients with birch pollinosis showed that YRC3780 ingestion could relieve allergy symptoms, decreased plasma thymus and activation-regulated chemokines and elevated plasma interferon-gamma levels. Therefore, it is possible that daily intake of YRC3780 boosts the immune system. If so, in the present study, the YRC3780 group might have experienced enhanced NK cell function and improved Th1/Th2 cell balance. It is plausible to assume that YRC3780 results in increased immune function that decreases the HPA axis response to chronic and acute stress (e.g., TSST).

With respect to the neural pathways involved from the gut to the central nervous system, 5-HT produced by interaction with intestinal chromaffin cells localized in the small intestine acts on 5-HT receptors of the vagus nerve or the of the spinal cord nerve terminal and transmits information to the brain [13]. Yoshikawa et al. [45] reported that ingestion of killed *L*. *brevis* SBC8803 cells stimulates 5-HT production and release from intestinal chromaffin cells in vitro and result in increased sleep quality in vivo. The immunomodulatory effects of YRC3780 result from its interaction with immune cells in the small intestine, which might be involved in 5-HT production, as in the *L*. *brevis* SBC8803 strain, which stimulates excessive production of inflammatory cytokines. Taken together, these findings indicate that the daily intake of YRC3780 alleviates chronic stress and acute stress in daily living conditions by regulating the immune system. Further studies are needed to evaluate whether the daily intake of YRC3780 could stimulate intraluminal 5-HT release from intestinal cells.

There are some limitations to the present study. The present study only examined healthy young male subjects. Previous studies have reported that the stress reactivity of the HPA axis to the TSST differs between males and females [46]. In addition, the stress reactivity of the HPA axis is altered by ageing [46]. Further studies are needed to assess whether daily intake of YRC3780 alters HPA axis functions in females and older subjects and to determine whether YRC3780 intake affects other stress-related hormones and hormone-releasing factors [47, 48]. In the present study, YRC3780 did not have an impact on the gut microbiome (Fig.4). However, the abundance of the microbiome is associated to the degree of anxiety and depression [49], which could be basis of the onset of the response to stress. Further studies might be needed to clarify the effect of YRC3780 on objective and subjective stress responses.

This study found that daily YRC3780 intake alters morning HPA axis basal activity, and improves subjective sleep quality, mental health, and HPA axis reactivity to acute psychological stress.

## Supplementary information

Supplementary material

Supplemental material

## Data Availability

The data that support the findings of this study are available from the corresponding author on reasonable request.
