# Correction to: Effects of *Lactococcus lactis* subsp. *cremoris* YRC3780 daily intake on the HPA axis response to acute psychological stress in healthy Japanese men

**DOI:** 10.1038/s41430-022-01153-y

**Published:** 2022-05-10

**Authors:** Noriko Matsuura, Hidemasa Motoshima, Kenji Uchida, Yujiro Yamanaka

**Affiliations:** 1 Laboratory of Life and Health Sciences, Faculty of Education and Graduate School of Education, Hokkaido University, Sapporo, Japan; 2 R&D Center, Yotsuba Milk Products Co., Ltd, Kitahiroshima, Japan; 3 Research and Education Center for Brain Science, Hokkaido University, Sapporo, Japan

**Keywords:** Randomized controlled trials, Sleep

Correction to: *European Journal of Clinical Nutrition* 10.1038/s41430-021-00978-3, published online 4 August 2021

In the original article the CFU of the test meal, YRC3780 powder, in the Supplementary Material was presented incorrectly.

Incorrect unit: 5.9 × 10^10^ CFU/g

Correct unit: 5.9 × 10^10^ CFU/250 mg

The Supplementary Material in the original article has been corrected.

