# Supplementary material for: Effects of Lactococcus lactis subsp. cremoris YRC3780 daily intake on the HPA axis response to acute psychological stress in healthy Japanese men
Source: Eur J Clin Nutr. 2021 Aug 4;76(4):574–80. doi: 10.1038/s41430-021-00978-3 (PMC8993685; doi:10.1038/s41430-021-00978-3)
Supplement: Supplementary file 1 — Supplementary material [file 41430_2021_978_MOESM1_ESM.docx]

**Supplementary Methods**

**Microbiological characterisation of *Lactococcus lactis* subsp. *cremoris* YRC3780**

The microbiological characterisation of YRC3780 was described in the patent (S1). YRC3780 has been deposited with the IPOD (International Patent Organism Depositary) and the accession number is FPRM P-18320. The main characteristics are as follows. (a) It was a gram-positive cocci, non-sporulating, catalase-negative, homofermentative, which produce L(+) lactic acid. (b) It does not grow at pH 9.2 and 9.6 in the initial pH test. In the salt tolerance test, neither 4% nor 6.5% grew. In the growth temperature test, it could grow at 10, 15, 25, and 32 °C, but it could not grow above 40 °C. (c) No ammonia production from arginine. (d) In the carbohydrate fermentation test using API50 CH (Japan Biomerieux Biotech), galactose, glucose, fructose, mannose, N-acetylglucosamine, cellobiose, and lactose were fermented. (e) In a partial sequence of the 16S rRNA gene (about 300bp including V1-V2 region), it showed >99% similarity to the Lactococcus lactis by homology search using Ribosomal Database Project. Additionally, PCR using species-specific primer (LcCr-F, Lc-R) (S2) was done, the specific band (product size: 551bp) was detected by electrophoresis. Lactococcus lactis have 4 subspecies (lactis, cremoris, hordniae, tructae) (S3), these results indicated that YRC3780 consisted of properties of Lactococcus lactis subsp. cremoris.

***Test meal***

The YRC3780 capsule contained YRC3780 powder (5.9×10^10^ CFU/250mg at the start of the study) and starch, and the placebo capsule contained only starch. The doses of YRC3780 were determined by reference to our previous study (S4) that reported an antiallergic effect of the YRC3780 intake in birch pollinosis patients. All participants received one capsule (approximately 250 mg) daily. Both capsules were brown in color, thus preventing identification of the contents. The capsules were stored in a refrigerator and kept in frozen during the intake period.

***Trier Social Stress Test***

Participants were instructed to visit our laboratory within two hours after waking up in the morning. The TSST was conducted between 7:00 and 10:30 am, because the HPA axis stress response to the TSST was reported to be higher in the morning than in the afternoon and evening (S5, S6). The experimental protocol of the TSST had a slight modification to the standard TSST protocol (S7). After arrival at the laboratory, participants rested for 20 minutes in room A (pre-stress period), then were placed in a second room (room B) by the experimenter, where a video camera was installed, and two trained experts were sitting at a table. The participant stood in front of the experts, who explained the first free speech task, then the participant returned to room A and was given 10 minutes to prepare their talk (anticipation period). At time +10 min, the participant was returned to room B by the experimenter and instructed to stand in front of the video camera and start free speech for five minutes, then performed mental arithmetic for five minutes (test period). Afterward, the participant returned to room A and rested for 30 minutes (post-stress recovery period). Saliva samples were taken for analysis of salivary cortisol concentrations at each phase of the TSST (20 min after arrival at the laboratory, 0 min after the anticipation period, and 0, 10, 20, and 30 min after the TSST). Beat-to-beat heart rate (HR) was monitored continuously throughout the TSST and averaged at each TSST phase.

***Saliva collection and salivary cortisol measurement***

Saliva samples were collected using a Salivette cotton swab (Sarstedt, Numbrecht, Germany), placed in the participant’s mouth for three minutes. After sampling, saliva samples were immediately frozen at -30°C until analysis. Salivary free cortisol concentrations were measured using a salivary cortisol ELISA kit (kit No. 1-3002, Salimetrics LLC, State College, PA, USA). The lowest detection limit of the assay was 0.33 nmol/L. Inter- and intra-assay variances were 3.0% and 2.6%, respectively.

***Microbiome analysis***

Microbiome analysis was basically performed according to the method as in previous study (S8). The V3-V4 region of 16S rDNA was amplified using Pro341F/Pro805R primer set (Table 2). Some modifications were described below. Feces from individual participants were collected using feces collection containers (TechnoSuruga Laboratory, Japan) and immediately frozen at -25°C until use. Sequencing was conducted using an Illumina MiSeq sequencing system (Illumina, San Diego, CA, USA) and MiSeq Reagent Kit version 3 (600 Cycle) chemistry. Analyses of sequence reads were performed using the Ribosomal Database Project Multiclassifier ver.2.11 (http://rdp.cme.msu.edu/classifier/). Bacterial identification was performed using the Metagenome@KIN analysis software (World Fusion, Japan) and the TechnoSuruga Lab Microbial Identification database DB-BA 13.0.

***Fecal sample collection and microbiome analysis***

Feces from individual subject were collected by feces collection container (TechnoSuruga Laboratory,Japan) and immediately frozen at under -25°C. All fecal samples were stored at -30°C until microbiome analysis. Frozen fecal samples were thawed on ice, 100 mg of each sample was suspended in 4 M guanidium thiocyanate, 100 mMTris-HCl (pH 9.0), and 40 mM EDTA, and the samples were then beaten with zirconia beads using a FastPrep FP100Ainstrument (MP Biomedicals, USA). DNA was extracted from the bead-treated suspensions using a Magtration System 12GC and GC series MagDEA DNA 200 (Precision System Science, Japan). Therefore, extracted DNA was purified using PI-480 (Kurabo Industries, Japan). DNA concentration was estimated by spectrophotometry using an ND-1000 instrument (NanoDrop Technologies, USA), and the final concentration of the DNA sample was adjusted to 10 ng/mL.

***Illumina library generation***

The V3-V4 region of 16S rDNA was amplified using Pro341F/Pro805R primer set for Prokaryotes and Bacteria (Table S1). Sequencing was conducted using a paired-end and modified to 2 × 300-bp cycle run on an Illumina MiSeq sequencing system (Illumina, San Diego, CA, USA) and MiSeq Reagent Kit version3 (600 Cycle) chemistry. Paired-end sequencing with read lengths of 301 bp was performed. After demultiplexing, a clear overlap in the paired-end reads was observed. The method of quality filtering of sequences was as follows: only reads that had quality value (QV) scores of ≥20 more than 99% of the sequences were extracted for further analysis.

***16S rDNA-based taxonomic analysis***

Analyses of sequence reads were performed manually using the Ribosomal Database Project (RDP) Multiclassifier ver.2.11, which is available from the RDP website (http://rdp.cme.msu.edu/classifier/). Reads obtained in the FASTA format were assigned to class levels with an 80% confidence threshold. Bacterial identification from sequences was performed using the Metagenome@KIN analysis software (World Fusion, Japan) and the TechnoSuruga Lab Microbial Identification database DB-BA 13.0 (TechnoSuruga Laboratory, Japan).

***Sleep measurement***

Participants’ sleep quality and quantity during the two-week baseline period were determined from the sleep diary and the Actiware-Sleep version 3.4 software (Minimitter, Bend, OR, USA), at one minute epochs, and medium sensitivity. We analyzed the mean Actiwatch-based sleep period time (SPT), total sleep time (TST), and percentage of sleep efficiency.

***Statistical analysis***

Statistical calculations were performed using a nonparametric test, since the salivary cortisol data were classified as not normally distributed by the Shapiro-Wilk normality test. Friedman tests and post-hoc Wilcoxon signed-rank tests were used for comparison with two values over the course of the experiment in the same group. The Mann-Whitney U test was used to compare the two groups. GraphPad Prism version 7 (GraphPad Software Inc., CA, USA) was used for all statistical analyses. Data are expressed as means ± SD. Figures are presented as means ± SEM.

**Supplemental references**

S1. Motoshima H, Uchida K. *Lactococcus* sp. isolated from kefir grains and food manufacturing methods using them. JP2001238579A. 2001-8-7.

S2. Odamaki T, Yonezawa S, Kitahara M, Sugahara Y, Xiao JZ, Yaeshima T, *et al*. Novel multiplex polymerase chain reaction primer set for identification of *Lactococcus* species. Lett Appl Microbiol. 2011; 52: 491–496.

S3. Pérez T, Balcázar JL, Peix A, Valverde A, Velázquez E, de Blas I, et al. *Lactococcus lactis* subsp. *tructae* subsp. nov. isolated from the intestinal mucus of brown trout (Salmo trutta) and rainbow trout (Oncorhynchusmykiss). Int J Syst Evol Microbiol. 2011;61:1894–1898.

S4. Uchida K, Motoshima H, Katano N, Hachimura S, Tanaka A, Nishihira J. Effect of Lactococcus lactis subsp. cremoris YRC3780 on birch pollinosis: a randomized, double-blind, placebo-controlled clinical trial. J Funct Foods. 2018;43:173–179.

S5. Kudielka BM, Schommer NC, Hellhammer DH, Kirschbaum C. Acute HPA axis responses, heart rate, and mood changes to psychosocial stress (TSST) in humans at different times of day. Psychoneuroendocrinology. 2004;29:983–992.

S6. Yamanaka Y, Motoshima H, Uchida K. Hypothalamic-pituitary-adrenal axis differentially responses to morning and evening psychological stress in healthy subjects. Neuropsychopharmacol rep. 2019;39:41–47.

S7. Kirschbaum C, Pirke KM, Hellhammer DH. The ‘Trier Social Stress Test’–a tool for investigating psychobiological stress responses in a laboratory setting. Neuropsychobiology. 1993;28:76–81.

S8. Takahashi S, Tomita J, Nishioka K, Hisada T, Nishijima M. Development of a Prokaryotic Universal Primer for Simultaneous Analysis of Bacteria and Archaea Using Next-Generation Sequencing. Plos One. 2014; 9: e105592.
