# Supplementary material for: Effects of Lactococcus lactis subsp. cremoris YRC3780 daily intake on the HPA axis response to acute psychological stress in healthy Japanese men
Source: Eur J Clin Nutr. 2021 Aug 4;76(4):574–80. doi: 10.1038/s41430-021-00978-3 (PMC8993685; doi:10.1038/s41430-021-00978-3)
Supplement: Supplementary file 2 — Supplemental material [file 41430_2021_978_MOESM2_ESM.docx]

| **Table S1. Primers** | |
| --- | --- |
| Primer name | Oligonucleotide sequence (5’-3’) |
| Pro341F | AATGATACGGCGACCACCGAGATCTACACTCTTTCCCTACACGACGCTCTTCCGATCTCCTACGGGAGGCAGCAG**CCTACGGGNBGCASCAG** |
| Pro805R | CAAGCAGAAGACGGCATACGAGATNNNNNNGTGACTGGAGTTCAGACGTGTGCTCTTCCGATCT**GACTACTACNVGGGAGGCAGCAG** |
| Underlined regions indicate Illumina adapter sequences. Bold-face font indicates PCR primer region preceded by a linker sequence. Poly-N string in forward primer denotes barcode sequence. Barcode sequences used in the present study (59-39): GATCTG, TCAAGT, CTGATC, AAGCTA, GTAGCC, TACAAG, CGTGAT, ACATCG, and GCCTAA. | |
